# An Up-Date of the Muscle Strengthening Exercise Effectiveness in Postmenopausal Women with Osteoporosis: A Qualitative Systematic Review

**DOI:** 10.3390/jcm10112229

**Published:** 2021-05-21

**Authors:** Jose Luis Alonso Pérez, Sebastián Martín Pérez, Andrea Battaglino, Jorge H. Villafañe, Alexandra Alonso-Sal, Eleuterio A. Sánchez Romero

**Affiliations:** 1 Musculoskeletal Pain and Motor Control Research Group, Faculty of Health Sciences, Universidad Europea de Madrid, 28670 Madrid, Spain; sebastian.martin@universidadeuropea.es (S.M.P.); alexandraalonsosal@hotmail.com (A.A.-S.); 2 Department of Physiotherapy, Faculty of Biomedical and Health Sciences, Universidad Europea de Madrid, Tajo, s/n, Urbanización El Bosque, Villaviciosa de Odón, 28670 Madrid, Spain; 3 Musculoskeletal Pain and Motor Control Research Group, Faculty of Health Sciences, Universidad Europea de Canarias, C/Inocencio García 1, Tenerife, Canary Islands, 38300 La Orotava, Spain; 4 IRCCS Fondazione Don Carlo Gnocchi, Piazzale Morandi 6, 20148 Milan, Italy; andrea.battaglino@edu.unito.it

**Keywords:** exercise, women, osteoporosis

## Abstract

Background: Osteoporosis (OP) is a systemic disease that is characterized by decreased bone density and quality. *Purpose*: The purpose of this systematic review was to determine the effects of muscle strengthening exercise in postmenopausal women with OP. *Methods*: A literature search was conducted systematically in MEDLINE, CINAHL, EMBASE databases for human studies up to 31 March 2021. Two researchers screened the articles against predefined inclusion criteria; a third resolved discrepancies. Articles were included if they assessed the effects of muscle strengthening exercise in postmenopausal women with OP. The protocol for this systematic review was registered on PROSPERO (CRD42021207917) and a qualitative systematic review was carried out following the PRISMA statement. Methodological quality was evaluated through the scientific validity scales PEDro. Finally, RTCs and NRCTs risk of bias was assessed with the Cochrane risk of bias tool (Risk of Bias-ROB 2.0) and ROBINS-1, respectively. *Results*: A total of 16 studies (1028 subjects) that met the different eligibility criteria previously established were selected. There is evidence of good methodological quality and a low to moderate risk of bias that supports that muscle strengthening exercise alone or in combination with other therapeutic modalities improves BMD (9, *n* = 401) in proximal femur and lumbar vertebra body, muscle strength (10, *n* = 558), balance (4, *n* = 159), functionality (7, *n* = 617), and quality of life (5, *n* = 291). Conclusions: Exercise programs focused on muscle strengthening have benefits for all variables studied in postmenopausal women with OP.

## 1. Introduction

Osteoporosis (OP) represents a pathology of important health implications, which identifies its clinical significance in the fracture that occur as a consequence of increased bone fragility [1]. In 2010, it was estimated that 22 million women aged over 50 years old in the EU had osteoporosis using the diagnostic criterion of the WHO [1]. The high societal and personal cost of osteoporosis pose challenges to publich health and physician, particularly since most patients with osteoporosis remain untreated [2].

The standard treatment is fundamentally pharmacological and aims to reduce the incidence of fractures through the interruption of the resolution mechanism [3]. It appears that although the benefits outweigh the risks, the association between the combination of sequential antiresorptive/anabolic pharmacotherapeutic cycles and the reduction of fracture risk using aminobisphosphonates, selective estrogen receptor modulators, denosumab and teriparatide has not been demonstrated. However, when combined with exercise this would help maintain bone architecture thanks to increased bone mass and muscle strength [4,5]. Muscle strengthening exercise would be effective because it is believed to increase muscle mass and decrease fat mass, especially in osteoporotic menopausal women in whom, by stimulating osteogenesis, it would reduce falls and fractures [6,7].

In contrast, Ashe et al. [8], Sanudo et al. [9] and Asikainen et al. [10] have respectively highlighted the lack of efficacy of resistance exercise in elderly women, pointing out a non-significant positive effect on body loss in resistance protocols alone and showing how even the combination of aerobic and resistance training does not lead to improvements in terms of BMD, muscle strength, flexibility and coordination, even in programs lasting more than one year. In addition, Benedetti et al. [4] has reported the low efficacy of long-term muscle strengthening exercise in elderly women in terms of cortical volumetric bone mineral density when performed with own body weight or resistance. Perhaps this is due to the diversity of the exercise used in the research detailed in the existing literature, and to the different nomenclatures used: physical exercise, exercise, resistance training, strength training, weight-bearing exercise training, resistance training programs, land exercise program, aquatic exercise program, physical activity program, circuit training, high-intensity resistance and impact training, adapted physical activity exercise protocol, progressive load training, strengthening exercises and multicomponent training.

Due to the diversity of studies found in the literature that clearly indicate the effects of muscle strengthening exercise on bone architecture, but also the existence of other studies that deny a positive effect, the purpose of this systematic review was to present an update of studies assessing the effects of muscle strengthening exercise in postmenopausal women with OP.

## 2. Materials and Methods

### 2.1. Data Source and Search Strategy

This is a systematic literature review of studies investigating or updating the effects of muscle strengthening exercise in postmenopausal women with OP Preferred Reporting Items for Systematic Review and Meta-Analyses (PRISMA) guidelines were followed during the design, search and reporting stages of this systematic review. The protocol for this systematic review was registered on PROSPERO (CRD42021207917).

### 2.2. Search Strategy

Independent researchers (J.L.A.P., S.M.P. and A.B.) conducted a qualitative systematic review following the PRISMA statement by introducing the keywords “Osteoporosis, Postmenopausal”, “Resistance Training”, “Exercise”, and “Isometric Contraction” as well as the free terms “Strength Training”, “Musculoskeletal”, “Exercise” and “Strength” combined with the Booleans “AND” and “OR” in metasearch engines Cochrane Library Plus and TripDataBase and electronic databases Pubmed (MEDLINE) and Physiotherapy Database (PEDro), Table 1, search equations.

### 2.3. Eligibility Criteria

Eligibility criteria were: (1) Randomized and non-randomized clinical trials, (2) published full text, (3) without restriction in language (4) between 1 January 2005 and 31 March 2021, (5) in which participate postmenopausal women with OP over 40 years (6) in an exercise program based on muscle strengthening exercise combined or not with other modalities.

### 2.4. Data Extraction

All relevant articles from the aforementioned datasets were identified by two reviewers who conducted the data extraction independently (J.L.A.P. and S.M.P.). A third author (A.B.) resolved discrepancies. Reviewers were not masked to any pieces of information regarding the authors, the journal or the outcomes for each article reviewed. A standardized form was used to extract data concerning study design, number and mean age of participants, year and country of publication, setting, exercise program involved, follow-up timing, clinical outcome measures and reported findings. The form was developed according to the directions of the Cochrane Handbook for Systematic Reviews of Interventions—Version 5.1.0. This form was pilot-tested for reliability using a representative sample of the studies to be reviewed.

### 2.5. Outcome Measure

The primary outcomes was the change of BMD at lumbar spine and femoral bone regions, muscle strength, balance, functionality and quality of life between baseline and follow-up.

### 2.6. Quality Assessment

All the articles that met the eligibility criteria were independently assessed by two independent authors (J.L.A.P. and S.M.P.) for methodological quality with the Physiotherapy Evidence Database (PEDro) Scale and for risk of bias using Cochrane Collaboration’s Risk of Bias (ROB.2.0) for RCTs and Risk of Bias in Non-randomized Studies of Interventions (ROBINS-1) for NRCTs. Disagreements were solved by discussion including a third author (A.B.) until a consensus was reached. We classified the methodological quality as follows: ≥ 7  =  high, 5–6  =  moderate, and <5  =  low and RCTS risk of bias as “high” risk of bias, “unclear” or “low” and NRCTs as “low”, “moderate”, “serious” and “critical”.

## 3. Results

### 3.1. Study Selection

**Figure 1:**
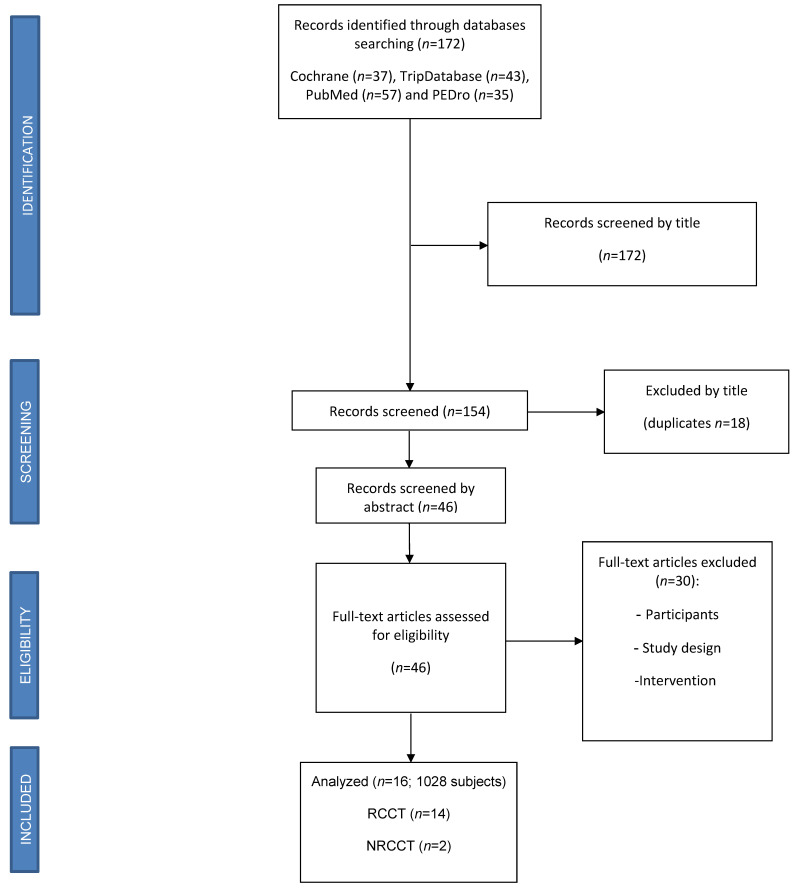
PRISMA Flow diagram.

Via the databases search, 172 articles have been identified. Of these, after first screening based on title, abstracts and duplicates, 46 articles have been submitted to a second screening. After the full text reading, according to out exclusion criteria, 16 studies were eligibility for this review, with a total amount of 1028 patient (Figure 1).

### 3.2. Study Characteristics and Quality Assessment

The review included 14 RCCTs and 2 NRCCTs, whose characteristics are collected in Table 1. The methodological quality evaluation, based on PEDro Scale, reports an average score of 6/10 (PEDro Table). The risk of bias analysis, using RoB 2.0 for RCTs and ROBINS-1 for NRCTs, showed a low to moderate risk and a critical risk respectively.

## 4. Summary of Results

### 4.1. Association between Exercise Therapy and Bone Quality

Nine of the 16 studies included in this review investigated the effects of exercise on bone quality. In the tested districts (femoral neck, lumbar spine, tibia) were found a statistically significant correlation between Bone Mineral Density and BMC with a 10 to 52 week exercise protocol (Table 1) [11,12,13,14,15,16,17,18,19]. However, Brentano and Ashe report absence of modifications and changes not statistically significant in this outcome measure [8,20].

### 4.2. Association between Exercise Therapy and Muscular Strength

In the included trials, muscle strength was evaluated at the level of back extensor strength, lower limb strength using leg press 45°, knee extension/flexion, ankle dorsiflexion, quadriceps strength (QS) and grip strength in the upper limb. The results agree in highlighting a significant correlation between an exercise protocol (Table 1) and muscle strength in all districts evaluated [14,15,23,24,26].

### 4.3. Association between Exercise Therapy and Balance

The dynamic balance was evaluated, within the trials, using the Tinetti Scale and the Berg Balance Scale; the static one was evaluated using a stabilometric platform (Stabilometric platform E.P.S./R/LorAn Engineering, Bologna, Italy), analyzing the stakinesiogram and the stabiligram. For both components (dynamic and static) a significant correlation between exercise and balance improvements was found [12,14,15,18,21,27], (Table 1).

### 4.4. Association between Exercise and Quality of Life

Koevska et al [22] and Cergel et al [23] investigated this outcome using QUALEFFO-41; Borba-Pinheiro et al [18] used in both studies the OPAQ questionnaire, an instrument to measure the quality of life in patients with low BMD levels. In the other two trials in which this outcome was evaluated, the EQ-5D questionnaire was used, with the addition, in the study of Marini et al [21] of the ECOS-16, a specific tool for osteoporosis in assessing quality of life.

With the exception of Koevska et al [22], who reported only statistically significant changes within the sample study groups, the results of the other authors agree on a significant correlation between the execution of an exercise protocol (Table 1) and improvements in quality of life compared to those who do not perform any exercise program.

### 4.5. Association between Exercise Therapy and Functionality

The evaluation of physical performance was measured using different types of tests: time up-and-go test, 5 time sit-to-stand, 6-min walking test, functional reach test (FRT), bend reach performance test (BRPT), vertical jump (VJ) and chair sit-and-reach; then SF-36 in its physical function component and Tinetti’s Scale were used.

The results report a statistically significant increase in performance in the evaluation tools used, with superiority of the groups performing an exercise protocol (Table 1) over the control groups [12,15,21,23,24,25,27].

## 5. Discussion

The purpose of this systematic review had to analyze the actual evidence about the muscle strengthening exercise and its efficacy in postmenopausal women suffering osteoporosis/osteopenia. The results of the studies analyzed, despite the wide range of years of publication, agree on the association between resistance exercise and its positive effect on the population examined in this review. However, the moderate-low level of methodological quality and the lack of homogeneity of the training programs analyzed suggest that there is contradictory evidence.

Strength training aims to promote osteogenesis in women diagnosed with OP, however, no significant changes are observed in vitamin-D levels, but significant changes are observed in bone architecture in both protein matrix and bone (*p* = 0.00177, *p* = 0.00031) as well as in BMD. It seems that these changes would be more pronounced if the strength programs had a duration of 12 months [28,29]. However, the studies included in the present review presented a wide range of exercise duration (from 12 weeks for the program of Mosti et al. to 13 months for the program of Borba-Pinheiro et al.) with inconsistency in the positive impact on bone quality, which does not allow a firm conclusion on the optimal durability of the programs [14].

Muscle strengthening exercise improves other capacities such as isometric and isotonic strength of large neuromuscular complexes of both the lower and upper limbs that seem to be key in the primary prevention of falls [30]. Furthermore, these improvements are related to work intensity, showing that interventions are required that work at least at an intensity of 80 to 85% 1RM to achieve the desired effects. Effects on balance are also observed, although these improvements do not seem to extend beyond 10 months, so we believe it is necessary that this ability be introduced as early as possible in training programs, especially if the intensity is high at a lower frequency. However, this last aspect has not been rigorously demonstrated because for many researchers it would be difficult to justify the use of high intensity in frail women diagnosed with OP To overcome this procedural obstacle, Watson et al. proposed in their LIFTMOR study to divide the program into two mesocycles of 6 months duration, avoiding that excessive load accumulation ends up putting these patients at risk [24].

Regarding the topic of exercise parameters, a 2020 review and meta-analysis by Shojaa et al., aiming to analyse the effects of dynamic muscle strengthening exercise on BMD in postmenopausal women [31], showed no significant difference in BMD between protocols with different duration of intervention and between different exercise intensities. On the contrary, it showed a significant difference with a positive effect on bone quality, in favour of training with free weights and a low net training frequency (<2 sessions/week).

On the other hand, it has been widely demonstrated that physical activity is able to promote bone formation, stimulating bone metabolism and its remodeling through mechanical loading (compression, tension and tissue shear) [32,33], improve hormonal regulation (estrogens, parathyroid hormone and glucocorticoids) [34,35,36,37] (with mimetic effect to hormone replacement therapy in postmenopausal women [38]), facilitate the regulation of signaling pathways [39,40,41,42,43,44], and stimulation of angiogenic-osteogenic responses [45]. However, and only from a clinical point of view, working in an aquatic environment may be a good approach to work in early phases due to the ease of working on the psychological and behavioral aspects associated with fear of movement.

Although not investigated in this review, considering the complexity and multifactorial nature of postmenopausal osteoporosis, further research is needed to investigate the possible synergistic effect of pharmacotherapy with certain exercise modalities, as highlighted in the study by Zhao et al. [46], who demonstrated that the combination of hormone replacement therapy (HRT) and a mixed-modality exercise protocol (high-impact activity in combination with high-intensity progressive muscle strengthening exercise was able to generate greater beneficial effects on hip and spine BMD in postmenopausal women than single-modality exercise.

With the results of this work, and given the absence of studies that address this question of clinical relevance, it is convenient to deepen the role of muscle strength training in the primary prevention of osteoporotic fracture in patients with osteopenia and even with delayed diagnosis of OP.

Furthermore, from a methodological point of view, differences have been detected in the proposed interventions, in the sample size and in the initial clinical status of the participants, which vary not only in issues such as age but also in the prognosis of their disease. Therefore, the existing variability and the lack of uniformity make it difficult to interpret and relate the different results, so it seems necessary to carry out more studies that assess suitability and promote the unification of criteria to achieve maximum effectiveness in therapeutic proposals to help us resolve the clinical question.

## 6. Conclusions

Muscle strengthening exercise in postmenopausal women with OP produces favorable results in terms of bone mineral density, strength, functionality, and quality of life. However, the benefits produced can be increased when combined with other therapeutic exercise modalities such as aerobic, balance and coordination.

## Figures and Tables

**Table 1:** Characteristics of included studies.

Author, Year	Participants	Outcome Measures	Intervention	Results	Conclusions	PEDro Score
Marini et. Al (2019) [21]	*n* = 44 women [22 experimental group (APA), 18 control group (CG)]. *Inclusion criteria:* Post-menopausal women Age: from 60 to 75 OP verified by dual energy X-ray absorptiometry With or without pharmacological therapy for OP One or more vertebral fractures verified by radiography	Health-Related Quality of Life ECOS-16 EuroQoL (EQ-5D-3L) Fear of Falling: FES-I questionnaire Lumbar Back Pain: - VAS - Physical performance: - POMA - 6-MWT - Chair Sit-and-Reach	Protocol duration: 6 months Frequency: 2 times per week Session: 1 h IG: Supervised -Warm-up: 15 min; multi-articular exercises, focus on joint mobilization, balance and postural control during walking. -Workout: 35 min; resistance bodyweight exercises (isometric and dynamic). -Cool down: 10 min; Stretching, Exercise in an upright and supine static position, holding a stretch position for up to 30 s. Drug exposure (% allocated subject): IG 100% (Bisphosphonates)	Adherence: 75.8% (56.4–97.8%) ECOS-16: APA group −0.5 ± 0.5, *p* = 0.001 *; CG +0.0 ± 0.3, ns EuroQoL VAS: APA +6.0 ± 16.6, ns; CG +1.9 ± 12.1, ns FES-I: APA −4.7 ± 7.4, *p* = 0.006 *; CG +0.9 ± 2.5, ns Lumbar back pain VAS: APA −1.2 ± 2.6, *p* = 0.029 *; CG +0.3 ± 3.3, ns Tinetti scale: APA +2.8 ± 5.2, *p* = 0.003 *; CG −0.7 ± 2.4. ns 6MWT: APA +52.2 ± 42.1, *p* < 0.001 *; CG −8.5 ± 45.2, ns Chair Sit-and-Reach right/left: APA +6.5 and +7.3, *p* = 0.002 */0.001 *; CG −0.6 and −0.2, ns * whitin group difference *p* < 0.05	The feasibility, the safety and the positive effect of the proposed exercise protocol on quality of life, fear of falling, balance and functional exercise capacity show that APA programs should be extended also to patients whit OP and a history of vertebral fracture.	6 /10
Koevska et al. (2019) [22]	*n* = 92 women *Inclusion criteria:* - Diagnosed OP - Total t-score −1,5 SD to −2.5 SD, determined with X-ray DXA densitometry.	Quality of life QUALEFFO-41	Protocol duration: 12 months Frequency: 3 times per week IG: Exercise and physical modalities (interferent currents and magnetic therapy for 3 week, each day with a weekend break). CG 1: Exercise CG 2: No exercise Exercise: 5 to 8 times -Respiratory -Strengthening of the paraverterbral muscles, upper and lower extremities muscles, abdominal muscle -Active exercise for maintaining the range of motion of the joints of upper and lower extremities and spine -Balance Drug exposure (% allocated subject): IG 100%; CG 100% (Bisphosphonates, calcium and vitamin D)	Pain: III vs. I, 59.3 ± 21.3 vs. 40.87 ± 20.6 (*p* = 0.004 *) III vs. II, 59.3 ± 21.3 vs. 31.0 ± 23.2 (*p* < 0.0001 *) Physical function: III vs. I, 41.8 ± 19.3 vs. 19.95 ± 13.3 (*p* < 0.0001 *) III vs. II, 41.8 ± 19.3 vs. 19.99 ± 15.4 (*p* < 0.0001 *) Social Life: III vs. I, 67.06 ± 27.9 vs. 34.8 ± 19.9 (*p* < 0.0001 *) III vs. II, 67.06 ± 27.9 vs. 27.65 ± 21.64 (*p* < 0.0001 *) Health perception: III vs. I, 78.2 ± 21.2 vs. 45.88 ± 22.1 ( *p* < 0.0001 *) III vs. II, 78.2 ± 21.2 vs. 41.5 ± 21.9 (*p* < 0.000 *) * whitin group difference *p* < 0.05	The exercise program for OP has significantly improved the quality of life in patients after one year of practicing in all four domains: pain, physical activities and mobility, social activities and perception about general health condition.	8/10
Çergel et al. (2019) [23]	*n* = 60 women *Inclusion criteria* - Established OP by means of dual-energy X-ray absorptiometry using WHO criteria for OP - History of one or more vertebral fractures verified by radiography - Aged 40–75 years old - In menopause at least 1 year Regular drug therapy for OP at least 6 months.	Spinal pain -VAS Back extensor muscle Strength - Hand dynamometer Trunk muscle endurance - Timed Loaded Standing test Thoracic kyphosis - Digital inclinometer Functional mobilty - Time Up and Go test (TUG) Health-related quality of life - QUALEFFO-41	Protocol duration: 6 weeks Frequency: 3 times per week IG: Supervised exercise group (SE) with full supervision of physiatrist. CG1: Home-based exercise group (HE) with instructional booklet and asked to apply the program at home. CG2: Daily life activities -Workkout: Back extensor *Strengthening exercises* In prone position: trunk extension, alternate arm raises, opposing arm and legs On the hands and knees position: opposing arm and leg raises. I-II weeks: 3 set of 8 rep III-IV weeks: 3 set of 10 rep V-VI weeks: 3 set of 12 rep Drug exposure (% allocated subject): IG 100%; CG 100% (Bisphosphonates) at least 6 months	VAS Rest: SE 2.80 ± 1.57 *, HE 5.15 ± 1.56 *, Control 5.75 ± 1.61 Activity: SE 2.75 ± 1.65 *, HE 5.85 ± 1.42, Control 6.30 ± 1.49 TUG (s): SE 8.5 ± 1.31 *, HE 12.10 ± 2.84 *, Control 12.40 ± 2.06 Back extensor strength (N): SE 45.2 ± 7.15 *, HE 38.5 ± 6.63 *, Control 34.75 ± 5.80 Trunk endurance (s): SE 108.05 ± 17.4 *, HE 56.80 ± 22.27 *, Control 47.10 ± 21.11 ** QUALEFFO-41: SE 32.48 ± 7.31 *, HE 44.32 ± 8.17 **, Control 45.44 ± 7.76 * *p* < 0.01 ** *p* < 0.05	This study demonstrated that short-term supervised back extensor strengthening training is superior to home-based program in terms of spinal pain, back extensor muscle stgrength, trunk endurance, functional mobility, and QoL for postmenopausal osteoporotic women with vertebral fractures.	6/10
Watson et al. (2019) [19]	*n* = 51 women *Inclusion criteria* - Women older than 58 years - Low bone mass (T-score < −1.0 at the hip and/or spine).	Magnitude of kyphosis: - DXA - Inclinometer and flexicurve Lateral vertebral assessment (LVA) and Vertebral body morphology: - DXA and Cobb angle	Protocol duration: 8 months Frequency: 2/week Session: 30 min Intervention group (HiRIT): Supervised Warm-up: 2 sets of deadlift at 50% to 7% 1RM First month: Body weight and low-load exercise variants, with focus on progressively learning the movement patterns. 4 fundamental exercise within 2 months Remainder intervention period: Resistance exercise (deadlift, overhead press, back squat) Training: 5 sets of 5 repetition Intensity: >80% to 85% 1 RM Drug exposure (% allocated subject): IG 100%; CG 100% (Bisphosphonates, calcium and vitamin D)	Height (cm): CON −0.1 ± 0.6, HiRIT +0.2 ± 0.6, *p* = 0.140 Inclinometer (°) - Relaxed standing: CON −4.2 ± 6.7 *, HiRIT −4.7 ± 6.3 *, *p* = 0.779 - Standing tal: CON −2.0 ± 8.1, HiRIT −5.3 ± 7.1 *, *p* = 0.167 Flexicurve kyphosis index (°): CON −1.9 ± 2.4 *, HiRIT −2.1 ± 2.2 *, *p* = 0.819 Cobb endplate angle (°): CON −0.6 ± 4.3, HiRIT +0.4 ± 4.4, *p* = 0.631 Cobb body angle (°): CON +0.5 ± 4.5, HiRIT −1.0 ± 4.5, *p* = 0.276 * whitin group difference *p* < 0.05	Observations from the LIFTMOR trial indicate that brief, twice-weekly, supervides HiRIT exercise for 8 months did not cause fragility fractures and improved thoracic kyphosis in postmenopausal women with low to very low bone mass.	7/10
Watson et al. (2018) [24]	*n* = 101 women Mean age: 65 ± 5 *Inclusion criteria* - Women older than 58 years - Low bone mass (T-score < −1.0 at the hip and/or spine).	Bone measures -Femoral neck BMD -Lumbar spine BMD -QUS -Calcaneal BUA -SOS -SI Physical performance -LES -BES -TUG -FTSTS -FRT -Maximal vertical jump test	Protocol duration: 8 months Frequency: 2/week Session: 30 min *IG:* Supervised First month: Body weight and low-load exercise variants, with focus on progressively learning the movement patterns. 4 fundamental exercise within 2 months Remainder intervention period: Resistance exercise: deadlift, overhead press, back squat Warm-up: 2 sets of deadlift at 50% to 7% 1RM Reps: 5 sets of 5 repetition Intensity: >80% to 85% 1 RM *CG*: Home-based 8-month, twice-weekly, 30-min Warm-up: walking (10 min) Cool down (5 min) Resistance exercise: low-load resistance training (lunges, calf raises, standing forward raise, and shrugs) Stretching: side-to-side neck stretch, static calf stretch, shoulder stretch, and side-to-side lumbar spine stretch. Reps: 10 to 15 Intensity: <60% 1 RM Drug exposure (% allocated subject): IG 100%; CG 100% (Bisphosphonates, calcium and vitamin D)	LS BMD (g/cm^2^): CON −1.2 ± 3.1%, HiRIT +2.9 ± 3.1 %, *p* < 0.001 * FN BMD (g/cm2): CON −2.0 ± 3.0 %, HiRIT +0.3 ± 3.0 %, *p* = 0.025 * BUA (dB/MHz): CON +0.8 ± 7.6 %, HiRIT +1.0 ± 7.6 %, *p* = 0.534 SI: CON +2.0 ± 6.8 %, HiRIT +2.7 ± 6.8 %, *p* = 0.200 SOS (m/s): +0.2 ± 1.1 %, HiRIT +0.3 ± 1.1, *p* = 0.006 * FN total BMC (g): CON −0.2 ± 23.6%, HiRIT +1.7 ± 23.7%, *p* = 0.077 FN total vBMD (g/cm^3^): CON −0.3 ± 24.3%, HiRIT +3.7 ± 24.3, *p* = 0.830 * whitin group difference *p* < 0.05	LIFTMOR trial showed a brief, supervised, twice-weekly HiRIT exercise intervention was efficacious and superior to previous programs for enhancing bone at clinically relevant sites, as well as stature and functional performance of relevance to falls in postmenopausal women with low to very low bone mass	7/10
Borba-Pinheiro et al. (2016) [14]	*n* = 52 women *Inclusion criteria* - Female volunteers -Aged over 50 years -Low BMD: T-score <1SD (low bone density) -Different ethnic population (descendants of Europeans, Blacks and Indians) -Patient being treated with sodium alendronate [70 mg] and/or vitamin D3 -No previous history of fractures -No history for at least 1 year of regular practice of physical activity -Indication/medical clearance for physical exercises practice.	BMD DXA Functional autonomy Latin American Development Group for Maturity (GDLAM): −10-m walk (10MW) -Rising from sitting position (RSP) -Rising frorm ventral decubitus position (RVDP) -Rising from a chair and walking around the house (RCWH) -Putting on and take off a shirt (PRTS) Muscular Strength 10 maximum repetitions test (10MR) Quality of life Osetoporosis Assessment Questionnaire (OPAQ)	Protocol duration: 13 months Session: 60 min *IG*: RT3 (3 times a week) CG: RT2 (two times a week) *Exercises:* leg press 45°; knee extension; plantar flexion; squats; hip adduction; gluts (machine for gluts); elbow flexion; elbow extension; shoulder adduction Posology: 3 sets per exercise. Repetitions numers/sets, rest intervals between exercises and sessions respected the scientific principle of inter-dependence volume x intensity (American College Sports Medicine). Intensity: between 60% and 90%; 7 months cycles (60%, 65%, 70%, 75%, 80%, 85% and 90%); in addition 3 bimonthly cycles (70%, 80%, 90%) Drug exposure (% allocated subject): IG 100%; CG 100% (Sodium alendronate [70 mg] and/or vitamin D3)	Total BMD: RT3 +0.10% * vs. CG +0.09%, *p* < 0.05 T2 +0.06% vs. CG, *p* = 0.046 Leg press 45°: RT3 * vs. RT2 * = +24.97% favorable to RT3 RT3/RT2 vs. CG = +84.1% / +59.1% favorable to RT3/RT2 Knee extension: RT3 */RT2 * vs. CG = +15.28% / +20.37% favorable to RT3/RT2 OPAQ total score: RT3 369.05 ± 19.9 *^,^§, RT2 348.8 ± 22.6 *^,^§^,^°, CG 311.4 ± 35.7 §^,^° * intra-group *p* < 0.05 § *p* < 0.05 inter-groups favorable RT3 ° *p* < 0.05 inter-groups faborable RT2	Both experimental groups presented favorable results for BMD, strength, FA and QoL. However, the RT3 showed the best results compared to other groups after 13 months of intervention.	8/10
Khalili et al. (2016) [25]	*n* = 183 women *Inclusion criteria* - Women with primary OP (with DEXA bone densitometry) - 30 to 50 degrees kyphosis (with lateral standard wiew thoracic spine x-ray graphs).	Quality of life - Persian version of the SF-36 QOL questionnaire	Protocol duration: 6 months Session: 30 min Frequency: 5 times a week IG Warm-up: Walk and back extensors Resistance exercise: Home-base Reps: 10 contractions of back extensor without increasing the low back lordosis Drug exposure (% allocated subject): IG 100%; CG 100% (Calcium [1000 mg], vitamin D [800 IU] daily, sodium alendronate [70 mg] weekly)	Phisical Component Score: Intervention group 270.55 ± 58.72 *, Control group 233.30 ± 67.47 *, *p* = 0.00 Mental Component Score: Intervention group 255.78 ± 61.19 *, Control group 239.58 ± 73.60, *p* = 0.39 * intra-group *p* < 0.05	Home-based exercise with no direct supervision improved QOL in elderly women whith OP at a 6-month follow-up.	8/10
Murtezani et al. (2014) [15]	*n* = 62 women *Inclusion criteria* Women recently diagnosed (within the past 6 months) with OP on account of a DEXA scan T score below −2.5 Aged 50–70 years No history of vertebral fractures or lower extremities fractures No endoprostheses or fixation materials Capable of signing written informed consent	Muscle Strength -GS -QS Flexibility - BRPT Balance - Berg Balance Scale (BBS) Gait time - 6MWT Pain - VAS	Protocol duration: 10 months Session: 55 min Frequency: 3 times a week IG (Land exercise) Warm-up: 10 min. Stretching and balance exercise at 70–80% *Exercises:* leg press 45°; knee extension; plantar flexion; squats; hip adduction; gluts (machine for gluts); elbow flexion; elbow extension; shoulder adduction Posology: 3 sets per exercise. Repetitions numers/sets, rest intervals between exercises and sessions respected the scientific principle of inter-dependence volume x intensity (American College Sports Medicine). Intensity: between 60% and 90%; 7 months cycles (60%, 65%, 70%, 75%, 80%, 85% and 90%); in addition 3 bimonthly cycles (70%, 80%, 90%) Drug exposure (% allocated subject): IG 100%; CG 100% (Dietary restriction and supplementation (Calcium [1000 mg] daily and Vitamin D [800–1000 IU] daily)	VAS: LE −81.26% *, Control −32.28%, *p* < 0.001 GS: LE −4.54% *, Control −2.35%, *p* = 0.002 QS: LE +4.4% *, Control +1.1% *, *p* = 0.002 BBS: LE +3.24% *, Control +3.04%, *p* = 0.38 6MWT: LE +18.72% *, Control +12.29% *, *p* < 0.001 BMD: LE +5.35% *, Control +3.92%, *p* < 0.001 T-score: LE −12.04% *, Control −6.44%, *p* < 0.001 * whitin group difference *p* < 0.05	Significant improvements in physical function and BMD suggest that land exercise is a possible alterative for postmenopausal women with OP.	6/10
Mosti et al. (2013) [11]	*n* = 16 women *Inclusion criteria* At least 2 years postmenopausal Age < 75 years old BMD t-score between −1.5 and −4.0 at the lumbar spine, femoral neck or total hip	MS, RFD and PF - Squat exercise machine BMC and BMD - Lumbar spine - Femoral neck - Total hip Vitamin D and Markers of Bone Metabolism Treadmill Testing - Peak oxygen consumption (VO_2_ peak)	Protocol duration: 12 weeks Frequency: 3 times a week IG: (Maximal Strength Training MST) Workout: supervised maximal strength training, focused on high acceleration during the concentric phase, resulting in a high RFD during muscle contraction. Posology: Each set was separated by 2–3 min rest. Intensity: If the participants could perform >5 repetitions, the training load was increased by 2.5 kg. Drug exposure (% allocated subject): IG 100%; CG 100% (Calcium and Vitamin D)	1RM (kg): TG 93.13 ± 8.10 *^,^°, CG 62.19 ± 14.36 Dynamic RFD (N/s): TG 1103.35 ± 282.75 *, CG 1386.02 ± 595.00 Peak force (N): TG 1397.23 ± 123.84 *, CG 1389 ± 260.00 BMC (g): TG lumbar +2.9 ± 2.8% (*p* = 0.012); femoral neck +4.9% ± 5.6% (*p* = 0.043), No change in CG Serum bone markers: - Vitamin D (nmol/L) TG 80.7 ± 29.2; CG 99.5 ± 16.5). P1NP and CTX no significant changes P1NP/CTX ratio TG +21.5 ± 40.5%, *p* = 0.093 * Difference within group, *p* < 0.05 ° Difference between group, *p* < 0.01	This study demonstrates that squat exercise MST, applying only one exercise, improves 1RM, RFD, and BMC in patients with OP and osteopenia.	6/10
Marchese et al. (2012) [12]	*n* = 22 women *Inclusion criteria* Diagnosis of osteopenia by DXA performed within 6 months Age between 40 and 80 years old	BMD - Lumbar spine - Proximal femoral epiphysis Osteocalcin and CTX in serum Electromyographic signal - Quadriceps femoris - Hip adductors - Adbominal - Paravertebral Static Balance - LC - MAO 6MWT Disability and Quality of Life - EuroQoL	Protocol duration: 24 weeks Frequency: 3 times a week Session: 60 min IG: Training group A combination of exercised designed to improve strength and muscle tropism, aerobic capacity, coordination and balance, designed to stimulate bone tissue in an atypical and intermittent compression, bending and tensile multi-directional stress. Drug exposure (% allocated subject): IG 18.1%; CG 27.2% (Antiresorptives)	Balance LC: TG −49.79%, CG +7.33%, *p* < 0.0001 * MAO: TG −45.92%, CG +0.33%, *p* = 0.002 * Muscle Strength (s-EMG, μV) Quadriceps femoris: TG +45.49%, CG −1.60%, *p* < 0.00001 * Adductors: TG +33.66%, CG −1.13%, *p* < 0.00001 Extensors of Trunk: TG +53.35%, CG −1.58%, *p* < 0.00001 * 6MWT: TG +33.33%, CG +16.18%, *p* < 0.0001 * EuroQoL Score: TG +34.52%, CG −12.30%, *p* = 0.0002 * BMD Lumbar spine TG +14.90%, CG −6.60%, *p* = 0.0005 * Hip TG +5.06%, CG −8.60%, *p* = 0.03 Markers CTX: TG −24.52%, CG +11.32%, *p* = 0.002 * Osteocalcin: TG −15.06%, CG +25.28%, *p* = 0.0003 * * whitin group difference *p* < 0.05	A improve strength and muscle tropism, coordination and balance, can provide advantages of unquestioned importance in bone mass, neuromuscular function, reduced risk of falling and general health.rehabilitation program of group exercise based on gravitational load, designed to	5/10
Burke et al. (2012) [26]	*n* = 33 women *Inclusion criteria* Women from 65 to 79 year of age Diagnosis of OP (according to the WHO criteria) BMD reduced at leat 2.5 SD compared with young adults (region of lumbar spine)	Postural control LOS CTSIBm Inferior Members Strength (Isometric Strength) Ankle dorsiflexion Knee extension Kn ee flexion	Protocol duration: 8 weeks Frequency: 2 times a week Session: 60 min IG (Strength group) Warm-up: 10 min walking at low intensity Exercises: Balance exercise (20 min): walking in the tandem position, on the tips the toes and heel, sideways, while raising the leg and controlateral arm; standing on one leg, in the tandem position; Strengthening exercises for lower limb (30 min): exercise for knee extensor muscle, hip flexors muscles and akle extensor muscles. Posology: 10 repetitions, 1 min between sets. CG1 (Stretching group) CG2 (Education) Drug exposure (% allocated subject): IG 94%; CG1 52%; CG2 56% (Medication and calcium supplementation)	Adherence: 82.3% Isometric strength: Ankle flexion IG +4.4 kg, CG +0.3 kg, *p* = 0.012 * Knee extension IG +4.43 kg, CG +0.1 kg, *p* = 0.003 * Knee flexion IG +1.71 kg, CG +0.22 kg, *p* = 0.003 * Postural control: COP velocity IG +2.34°/s, CG 0.01°/s, *p* = 0.009 * Directional control IG +5.34 %, CG 0.44 %, *p* = 0.002 * CTSIBm (closed eyes) IG −0.21°/s, CG +0.05°/s, *p* = 0.021 * * whitin group difference *p* < 0.05	Our study suggests that, in old woman with OP, 8 weeks of exercises improving balance and inferior member strength yielded improvement of postural control and of muscular strength.	6/10
Borba-Pinheiro et al. (2010) [18]	*n* = 28 women *Inclusion criteria* Women with OP and/or osteopenia in at least one of the measurements of BMD T-score Patients treated with sodium alendronate (70 mg) No history of fractures No history for at least 1 year of regular practice of physical activity Good physical and mental health	BMD - Lumbar spine - Proximal femur Body balance - Static Balance Test with Visual Control Quality of Life - OPAQ	Protocol duration: 12 months Frequency: 3 times a week Session: 60 min IG: RTG Exercises: leg press 45°; knee extension; plantar flexion; squats; hip adduction; gluts (machine for gluts); elbow flexion; elbow extension; shoulder adduction Posology: 10 maximum repetitions (10RM) test Intensity: 70–90% CG1: JUG Exercises: Traditional methodology for judo classes CG2: WAG Exercises: in a 25-m pool, 1.45 m deep; dislocations (previous, posterior and lateral), shoulder adduction/abduction, short jumps with knee extension, alternate elbow flexion, alternate knee flexion, alternate elbow extension, hip adduction/abduction, shoulder abduction/adduction, squats. Drug exposure (% allocated subject): IG 100%; CG 100% (Sodium alendronate [70 mg] weekly)	BMD Lumbar: RTG 0.091, JUG 0.079, WUG 0.034, CG −0.024, *p* = 0.002, *p* = 0.003, ns Neck of femur: RTG 0.083, JUG 0.019, WUG −0.007, CG −0.06, *p* = 0.002, ns, ns Great trochanter: RTG 0.049, JUG 0.015, WUG, 0.018, CG −0.029, *p* = 0.002, ns, ns Body balance RTG 5.74, JUG 5.30, WUG 0.018, CG −1.06, *p* = 0.018, *p* = 0.022, ns OPAQ RTG 30.56, JUG 53.09, WUG 7.63, CG −7.29, *p* = 0.006, *p* = 0.000, ns * whitin group difference *p* < 0.05	The type of physical activity examined in this study could be raccomended alone or as adjunvtive therapy to a biphosponate in postmenopausal women with low BMD, especially resistance training.	5/10
Teixeira et al. (2010) [27]	*n* = 100 women *Inclusion criteria* - Aged from 55 to 75 years old - Individuals with postmenopausal OP. - BMD T-score of −2.5 SD in the lumbar spine, femoral neck or total femur region	Quality of life SF-36 Functional mobility - TUG Balance Berg Balance Muscular strength - Dynamic strength of the quadriceps muscle (1-RM)	Protocol duration: 18 weeks Frequency: 2 times a week IG Warm-up: 5–10 min treadmill, static stretching exercises (global and segmentary) for upper and lower limbs, lumbar, cervical, and thoracic region; 2 series of 3 rep for each muscle; 30 s maintening. Workout: Functional exercises (proprioception and balance) Strengthening exercises included leg extension, load up to 80% 1RM (following a two week protocol, from 50% to 80%) Drug exposure (% allocated subject): IG 100%; CG 100% (Antiresorptives)	SF-36: Δ in all subscales > 13.5 points, *p* ≤ 0.0018 Berg Scale: Δ 3.58 [2.75;4.42], *p* < 0.0001 Maximum load (kg): Δ 3.65 [2.74;4.57], *p* < 0.0001 Time Up and Go test (s): Δ −3.96 [−4.63; −3.29], *p* < 0.0001	The progressive muscle strength training for the quadriceps associated to the proprioceptive training is effective in increasing muscle strength in quadriceps, improvement in static and dynamic balance, speed of the motor responses, therefore improving the performance of daily activities and reducing the frequency of falls in women with postmenopausal OP.	6/10
Bocalini et al. (2009) [17]	*n* = 35 women *Inclusion criteria* Women older than 55 years Able to train 3 times per week in the course of 24 weeks of the protocol	Body composition BMI Body fat percentage BMD - Lumbar spine - Femur neck Muscle Strength (1RM) - Chest press - Leg extension	Protocol duration: 24 weeks Frequency: 3 times a week Session: 1 h supervised IG Warm-up: 10 min of running with low impact at 50% of maximum hearth rate; 1 set at 50% 1RM Workout (TR): Focus on eccentric muscle action. Leg press, chest press, leg curl, latissumus pull down, elbow flexion, elbow extension, leg extension, upper back row, military press, hip abductor, hip adductor, abdominal curls. Drug exposure (% allocated subject): IG 100%; CG 100% (Antiresorptives)	MS: TR 62 ± 5 kg, +39%, *p* < 0.001 lower limb; 37 ± 6 kg, +46%, *p* < 0.001 upper body UN 38 ± 7 kg, −2.5%, *p* > 0.05 lower limb; 23.5 ± 5 kg, +4.5%, *p* > 0.05 upper body BMD: TR 0.880 ± 0.001 g/cm^2^, *p* > 0.05 lumbar spine, 0.704 ± 0.001 g/cm^2^ femoral neck UN 0.873 ± 0.002 g/cm^2^, *p* < 0.05 lumbar spine, 0.695 ± 0.001 g/cm^2^ femoral neck	We demonstrated the positive effects of strength training on the body composition parameters, muscular strength, and bone health of postmenopausal women without hormone replacement therapy.	6/10
Tolomio et al. (2008) [16]	*n* = 64 women *Inclusion criteria* Postmenopausal women (age between 50 and 70 years) Diagnosis of osteopenia or OP (*t*-score determined by ultrasounds < 1.0SD Lack of any disease that affect bone metabolism No previous skeletal fractures Lack of any controindication to perform physical activity	Bone quality Phalangeal quantitative osteosonography As-so UBPS Muscle Strength (1RM) - Knee extensor muscles	Protocol duration: 20 weeks Frequency: 3 times a week Session: two 60-min sessions and one 45-min session IG *60-min session:* Warm-up: 20–25 min of walking, stretching, small jumps. Workout: 30-min training; callistheni/isometric exercises and exercises with dumbells, Thera-Bands, balls aimed to improve range of motion, overall Strength, balance and aerobic capacity. Cool down: 5–10 min; stretching, breathing, postural exercises Volume: graded increase of intensity and number of rep/series starting after the fifth week of training. CG *45-min session:* Combination of aerobic endurance and Strength exercises. Workout: Circuit training of 6 bouts of exercise lasting 5 min each; treadmill, leg extension, arm ergometer, horizontal leg press, bike, lat machine. Indication to progressively increase repetitions or load lifted in during each 5-min Strength exercise. Drug exposure (% allocated subject): IG 58,6%; CG: 55% (Bisphosphonates, calcium and Raloxifene)	Ad-Sos: EG 1988.8 ± 74.4 m/s, *p* < 0.05; CG ns UBPS: EG 36.8 ± 21.3, *p* < 0.05; CG 36.5 ± 17.2, ns T-score: EG −2.1 ± 1.1, *p* < 0.05; CG ns Knee extension: 52.7 ± 9.5 kg, *p* < 0.05; CG ns	In a group of postmenopausal women, a supervised, multidimensional exercise program improved bone quality, evaluated at the finger, in a relatively short period of time.	6/10
Brentano et al. (2008) [20]	*n* = 28 women *Inclusion criteria* No neuromuscular injury or engaged in any tipe of competitive exercise Practiced sports occasionally at a recreational level.	Body composition BM FFM FM SF VO_2_ max TE Dynamic Strength (1RM) Arm curl exercises Knee extension exercises Isometric Strength MVC Electromyographic Signal Vastus lateralis Vastus medialis BMD Lumbar spine Femur	Protocol duration: 24 weeks Frequency: 3 times a week Session: 1 h supervised Warm-up: 5 min; cycloergometer or treadmill Workout: leg press, hip abduction, hip adduction, knee extension, chest fly, reverse fly, arm curl, triceps push-down, sit-ups, back extension. *IG: STG* The exercises were performed separately, with a 2-min rest between sets. Posology: 20–6 repetitions and 45–80% 1RM, 2–4 sets for each exercises. *CG: CTG* The exercises were performed with no rest between exercises Posology: 23–10 repetitions and 45–60% 1RM; 2–3 sets for each exercise. Drug exposure (% allocated subject): IG 50%; CG: 50% (Hormone therapy (HT))	VO_2_max and TE: increased significantly in both training group after 24 weeks Dynamic strength: LDS and UDS increased significantly in STG and CTG, greater than the CON group. Isometric strength: Increased significantly in both training group after 24 weeks BMD: no alteration in BMD lumbar, BMD neck, BMD inter, BMD troc, BMD ward in all groups after the 24-week period. Correlations: LLS and VO_2_max: *r* = 0.73, *p* = 0.000 LLS and TE: *r* = 0.72, *p* = 0.000 IS and VO_2_max: *r* = 0.59, *p* < 0.01 IS and TE: *r* = 0.54, *p* < 0.01	Circuit weight training is an effective training in strategy to improve neuromuscular and cardiorespiratory conditioning of postmenopausal women with no history of resistance training.	6/10

APA: Adapted Physical Activity; ECOS-16: Assessment of health related quality of life in OP questionnaire; EuroQoL: Euro Quality of Life; FES-I: Falls Efficacy Scale International; HRQOOL: Health-related quality of life; HiRIT: High-intensity, progressive resistance and impact training; LIFTMOR: Lifting Intervention For T raining Muscle and Osteoporosis Rehabilitation; OP: Osteoporosis; POMA: Tinetti Performance-Oriented Mobility Assessment tool; QUALEFFO-41: Quality of Life Questionnaire; RM: Repetition maximum; 6MWT: Six minute walking test; TUG: Time Up and Go test; VAS: Visual Analogue Scale; X-ray DXA: Dual-energy X-ray absorptiometry; AE: Aquatic Exercise; BMD: Bone Mineral density; BES: Back extensor strength; BRPT; Bend reach performance test; BUA: Broadband ultrasound attenuation; FN BMD: Femoral Neck Bone Mineral Density; DXA: dual-energy x-ray absorptiometry; FTSTS; 5 times sit-to-stand test; FN total BMC: Femoral Neck total Bone Mineral Component; FN total vBMD: Femoral Neck total velocity Bone Mineral Density; FRT: Functional reach test; GS: Grip strength; HiRIT: High-intensity, progressive resistance and impact training; LE: Land Exercise LES: Lower limb extensor strength; LS BMD: Lumbar spine Bone Mass Density; OPAQ: Osteoporosis Assessment Questionnaire; OP: Osteoporosis; QS:Right quadriceps strength; QUS: Quantitative ultrasound; SI: Stiffness index; SOS (m/s) Speed of sound; 6MWT: Six minute walking test; TUG: Time up-and-go test; VAS: Visual Analogue Scale; BMC: Bone Mineral Content; BMD: Bone Mineral density; COP: center of pressure; CTSIBm: Modified clinical test of sensory interaction for balance; CTX in serum: Carboxy-terminal collagen crosslinks (CTX) in serum; EuroQoL: Euro Quality of Life; JUG: Judo Group; LC: Lenght of clew; LOS: Limit of stability test; MS: Maximal strength; MAO: Maximum Amplitude of Oscillation; OPAQ: Osteoporosis Assessment Questionnaire; OP: Osteoporosis; PA: physical activity; PF: Peak force; P1NP; procollagen amino terminal peptides; RFD: Rate of Force Development; RT: resistance training; RTG: resistance training Group; 6MWT: Six minute walking test; WAG: Water Group; WHO: World Health Organization; As-sos: Amplitude-dependent speed of sound through the bone; BM: Body mass; BMD: Bone Mineral density; BMI: Body mass index; CTG: Circuit training group; CWT: circuit weight training; FM: Fat mass; FFM: Fat-free mass; IS: Isometric strength; LLD: lower limb dynamic strength; MS: Maximal strength; MVC: M aximal voluntary contraction; OP: Osteoporosis; RM: Repetition maximum; SF: Skinfolds sum; SF-36: Short Form Health Survey; ST: traditional high-intensity strength training; STG: Strength training group; TE: Time to Exhaustion in Exercise; TR: Trained Group; TUG: Time up-and-go test; UBPS: Ultrasound bone profile score; UN: Untrained GrouU+0070.

## Data Availability

The data presented in this study are available on request from the corresponding authors.
